# The Personalized Parkinson Project: examining disease progression through broad biomarkers in early Parkinson’s disease

**DOI:** 10.1186/s12883-019-1394-3

**Published:** 2019-07-17

**Authors:** B. R. Bloem, W. J. Marks, A. L. Silva de Lima, M. L. Kuijf, T. van Laar, B. P. F. Jacobs, M. M. Verbeek, R. C. Helmich, B. P. van de Warrenburg, L. J. W. Evers, J. intHout, T. van de Zande, T. M. Snyder, R. Kapur, M. J. Meinders

**Affiliations:** 1 Department of Neurology, Donders Institute for Brain, Cognition and Behaviour, Radboud University Medical Center, Nijmegen, The Netherlands; 2 Verily Life Sciences, South San Francisco, CA USA; 3 CAPES Foundation, Ministry of Education of Brazil, Brasília/DF, Brazil; 4 Department of Neurology, Maastricht University Medical Center, Maastricht, The Netherlands; 5 Department of Neurology, Universtity Medical Center Groningen, Groningen, The Netherlands; 6 Faculty of Science, University of Nijmegen, Nijmegen, The Netherlands; 7 Department of Neurology, Donders Institute for Brain, Cognition and Behaviour, Donders Center for Medical Neuroscience, Radboud University Medical Center, Nijmegen, The Netherlands; 8 Department of Laboratory Medicine, Donders Institute for Brain, Cognition and Behaviour, Donders Center for Medical Neuroscience, Radboud University Medical Center, Nijmegen, The Netherlands; 9 Institute for Computing and Information Sciences, Radboud University, Nijmegen, The Netherlands; 10 Department for Health Evidence, Radboud Institute for Health Sciences, Radboud University Medical Center, Nijmegen, The Netherlands; 11 Department of Neurology, Radboud University Medical Center, Nijmegen, The Netherlands; 12 Neurology Platform, Verily Life Sciences, South San Francisco, CA USA; 13 Scientific Center for Quality of Healthcare, Radboud Institute for Health Sciences, Radboud University Medical Center, Nijmegen, the Netherlands

**Keywords:** Cohort studies, Parkinson’s disease, Biomarkers, Disease progression, Wearable device

## Abstract

**Background:**

Our understanding of the etiology, pathophysiology, phenotypic diversity, and progression of Parkinson’s disease has stagnated. Consequently, patients do not receive the best care, leading to unnecessary disability, and to mounting costs for society. The Personalized Parkinson Project (PPP) proposes an unbiased approach to biomarker development with multiple biomarkers measured longitudinally. Our main aims are: (a) to perform a set of hypothesis-driven analyses on the comprehensive dataset, correlating established and novel biomarkers to the rate of disease progression and to treatment response; and (b) to create a widely accessible dataset for discovery of novel biomarkers and new targets for therapeutic interventions in Parkinson’s disease.

**Methods/design:**

This is a prospective, longitudinal, single-center cohort study. The cohort will comprise 650 persons with Parkinson’s disease. The inclusion criteria are purposely broad: age ≥ 18 years; and disease duration ≤5 years. Participants are followed for 2 years, with three annual assessments at the study center. Outcomes include a clinical assessment (including motor and neuro-psychological tests), collection of biospecimens (stool, whole blood, and cerebrospinal fluid), magnetic resonance imaging (both structural and functional), and ECG recordings (both 12-lead and Holter). Additionally, collection of physiological and environmental data in daily life over 2 years will be enabled through the Verily Study Watch. All data are stored with polymorphic encryptions and pseudonyms, to guarantee the participants’ privacy on the one hand, and to enable data sharing on the other. The data and biospecimens will become available for scientists to address Parkinson’s disease-related research questions.

**Discussion:**

The PPP has several distinguishing elements: all assessments are done in a single center; inclusion of “real life” subjects; deep and repeated multi-dimensional phenotyping; and continuous monitoring with a wearable device for 2 years. Also, the PPP is powered by privacy and security by design, allowing for data sharing with scientists worldwide respecting participants’ privacy. The data are expected to open the way for important new insights, including identification of biomarkers to predict differences in prognosis and treatment response between patients. Our long-term aim is to improve existing treatments, develop new therapeutic approaches, and offer Parkinson’s disease patients a more personalized disease management approach.

**Trial registration:**

Clinical Trials NCT03364894. Registered December 6, 2017 (retrospectively registered).

## Background

Parkinson’s disease (PD) is the second most prevalent degenerative brain disease [1]. PD will become even more prevalent in our aging society, with an expected pandemic rise and doubling of the current patient volume by the year 2040 [2]. From a systems-biology perspective, PD is seen increasingly as an umbrella term for a group of underlying disorders that all exhibit unique genetic, biological, and molecular abnormalities, and that are likely to respond differentially to a given therapeutic approach [3]. Although adhering to the concept of PD as a single disorder has been useful for the development of symptomatic treatments, it has hindered progress when applied to the development of new interventions that can decelerate or even arrest disease progression [4]. Moreover, our understanding of the basic etiology, pathophysiology, phenotypic diversity, and progression of PD has stagnated, partly due to the limited patient diversity captured in study cohorts, and partly because patients were not studied in sufficient detail (i.e., limited set of outcomes, or brief follow-up periods). As a result, patients do not receive the best care they deserve, leading to unnecessary disability and to mounting costs for society.

To date, there are no reliable biomarkers that can help to predict the widely varying differences between patients in prognosis, rate of progression, time to development of important milestones (e.g., time to first fall, or time to onset of dementia), or treatment response. Cohort studies evaluating multiple outcomes simultaneously are needed to address the multi-dimensional nature of PD. Although several of such cohorts exist and have already shown their merits [5–12], they all have their limitations. These limitations relate to, e.g., the inclusion of a highly selective patient population (rather than a more real-life population), to the multi-center design (introducing variability in assessments and procedures across centers), the lack of detailed clinical and genotyping baseline and follow-up information, the lack of repeated and advanced neuro-imaging, and the absence of wearable sensors to support the follow-up period.

The Personalized Parkinson Project (PPP) intends to build a cohort that has unique characteristics to overcome the limitations of previous studies. The project will focus on the inclusion of “real world” representative patients with PD, who will undergo extremely detailed phenotyping, including microbiome & inflammation, high-quality repeated neuroimaging with repeated MRI scans and fMRI. All assessments are performed at a single centre and optimal retention strategies will be applied. In addition, we seek to perform day-to-day patient monitoring with a multi-sensor wearable device that allows continuous real-time data collection in the free-living environment during a 2-year follow-up period using a state-of-the-art and convenient wearable device. The PPP aims to identify biomarkers that can assist in predicting differences in prognosis and treatment response between patients. The insights generated by the PPP will pave the way to improve existing treatments, to create new therapeutic approaches, and to develop a more precise and personalized disease management approach. Additionally, the PPP cohort applies unprecedented digital security standards, supporting data sharing with qualified researchers worldwide, allowing them to add their research capacity to further address the main aims of this study.

## Methods/design

### Study design

This is a prospective, longitudinal cohort study involving 650 persons with PD. During a 2-year follow-up period, participants will undergo three extensive annual assessments, and continuously wear a multi-sensor investigational research device (the Verily Study Watch). The 5-year study is being carried out in one academic hospital in the Netherlands (the Radboud university medical center in Nijmegen). Participants will be included between October 2017 and December 2019, or longer until the recruitment target of 650 participants is achieved.

Throughout the study period, a panel of 20 Parkinson patients will advise the research team on aspects of the study. For example, during the preparation phase, the panel advised on the design of the study procedures, on recruitment materials, and on subject retention strategies.

### Study population

Participants are eligible for this study if they meet the following criteria:

- Parkinson’s disease duration ≤5 years, defined as time since the diagnosis made by a neurologist;
- 18 years of age or older;
- Able to read and understand Dutch;
- Willing, competent, and able to comply with all aspects of the protocol, including follow-up schedules and biospecimen collections; and
- Providing written informed consent.

Patients with co-morbidity are explicitly NOT excluded from participation. Candidates will only be excluded in the case of existing co-morbidities that are sufficiently severe so as to hamper the interpretation of parkinsonian disability. Other exclusion criteria include contraindications to magnetic resonance imaging (MRI), pregnancy or breastfeeding, and/or nickel allergy (as components of the Study Watch contain this metal). For the lumbar puncture additional contraindications are defined, i.e., being treated with an anticoagulant medication or clinical evidence of structural cerebral abnormalities.

To guarantee a balanced study cohort, a stratification inclusion model will be applied, by gender (men/women), age (21–45;46–55; 56–65; ≥66 years) and disease duration (< 2½ years; ≥2½ years). Consequently, half of the cohort will have a disease duration of maximally 2½ years at the moment of inclusion, which will also allow us to the impact of very early signals and markers on disease progression.

### Recruitment and enrolment

In the Netherlands, approximately 40,000 people are diagnosed with PD, with an incidence of 8,000 newly diagnosed patients annually. Recruitment strategies that have proven to be successful in past clinical trials will be adopted [13–16]. First of all, the Dutch national ParkinsonNet, an existing nationwide clinical infrastructure in the Netherlands with 3,200 specialized PD professionals, will bring the study to the attention of its members [17–19]. Next, healthcare providers working in university medical centers and community-based hospitals treating PD patients and the Dutch Parkinson Patient Association, representing over 8,000 Dutch Parkinson patients, will promote the study. Finally, various social media announcements and media appearances will be used to reach the target population. All of those interested in the study will be redirected to the study website (www.parkinsonopmaat.nl), where detailed information can be found, along with an application form.

After application, participants receive at least two personal sessions with a dedicated trial assessor by phone to screen eligibility and provide further information. During these calls, prior to inclusion, the assessor verifies that the participant is competent, by discussing the participant’s decision and reasoning. The assessor can decide to not include a potential participant because of limitations in cognitive status that would keep the person from providing informed consent. If the patient is willing and able to participate, (s)he is invited to return a signed informed consent form by mail to the study center and the date for the baseline visit is booked. Once informed consent is obtained, access to the medical records of the treating neurologist and general practitioner is requested to verify the diagnosis of Parkinson’s disease and check whether or not patients are diagnosed with clear dementia in the eyes of the patient’s own physician. Those participants without a confirmed diagnoses and/or presence of dementia, will not be included, despite initial consent. During the first study visit, prior to the assessments, the trial assessor reconfirms that the patient understands the consequences of participation. During the follow-up visits the assessor will verify the cognitive status of the participants in a similar manner. The informal caregiver can also signal a marked cognitive decline that would hamper the patient’s inability to understand the implications of continued participation. Patients who are no longer able to provide a valid informed consent for participation will be excluded from further follow-up, but the data obtained until that moment will be kept in the central database.

### Study procedures and assessments

Participants visit the study site three times for data collection: an initial baseline visit and two annual follow-up visits. Data collection during each visit takes a full day and can be spread out over a 1.5-day period if desired by the patient. Prior to each visit, study participants will be offered a complimentary hotel stay, thereby reducing the need for patients living far away from the study center to travel long distances in the early morning. Patients arrive at the study site in the morning in a practically defined OFF state, i.e., at least 12 h after having taken their last dopaminergic PD medication.

During the three in-clinic visits, detailed clinimetrics with multiple standardized evaluations for motor, neuropsychological, and other PD-related assessments are performed, while wearing the Verily Study Watch (see Table 1 for an overview). Some of the motor assessments are performed first in a clinically defined OFF state and repeated after the participant has taken the regular first morning dose of medication(s) and has subjectively reached a typical ON state. The medical examination and clinical assessments are videotaped with a static camera and/or recorded for audio signals, unless the subject declines. The video-recording are for local use only and will not be shared within the study. In addition, relevant biospecimens are collected, i.e., whole blood for plasma/peripheral blood mononuclear cells (PBMC)/serum/DNA/RNA, cerebrospinal fluid (CSF), and stool for longitudinal genotypic and phenotypic assays. CSF will be collected only at baseline and after 2 years and is the single optional element of the study protocol. Local anaesthesia will be provided to minimize patient burden and atraumatic needles will be used. Moreover, using advanced MRI, at baseline and after two years, indices of in vivo brain structure and function are obtained, using an extension of the UK Biobank protocol [20, 21]. The PPP MRI protocol captures structural MRI (T1 and T2-weighted imaging, diffusion tensor imaging (DTI) and quantitative susceptibility mapping (QSM)), resting functional MRI (fMRI), and two task-related fMRI studies. The tasks probe both primary brain dysfunction, i.e. basal ganglia dysfunction, as well as cerebral compensatory activity. An additional surface electromyography is performed during MRI scanning to monitor tremor, so artifacts can be removed [22]. Finally, to assess autonomic dysfunction, study subjects wear a Holter monitor during each in-clinic visit (except while being in the MRI scanner), and a 12-lead ECG is performed while the subject is supine. Blood pressure is measured in both supine and standing positions, from which the presence of orthostatic hypotension will be assessed.

**Table 1:** Overview of included study measures and scales in the Personalized Parkinson Project

Method	Outcome	Scales	Visit 1 (Baseline)	Visit 2 (1 Year)^b^	Visit 3 (2 Years)^b^	After each visit^c^
Assessed by trial assessor	Motor functioning in OFF^a^ state	- MDS-UPDRS-III (including H&Y stage)	X	X	X	
		- Timed up-and-go test	X	X	X	
		- Standing leg test	X	X	X	
		- Rapid turning test	X	X	X	
		- Pegboard test	X	X	X	
	Motor functioning in ON^a^state	- MDS-UPDRS-III (including H&Y stage)	X	X	X	
		- TUG	X	X	X	
		- MDS-UPDRS-IV	X	X	X	
		- Standing leg Test	X	X	X	
		- Rapid Turning Test	X	X	X	
		- Pegboard Test	X	X	X	
		- Grip strength	X	X	X	
	Neuropsychological symptoms	- MDS-UPDRS-I	X	X	X	
		- MoCa (with alternative versions during follow-up)	X	X	X	
		- Phonemic and semantic Fluency	X	X	X	
		- Brixton Spatial Anticipation Test	X	X	X	
		- 15 Words Test	X	X	X	
		- Benton Judgment of Line Orientation	X	X	X	
		- Letter Number Sequencing	X	X	X	
		- Symbol Digit Modalities Test	X	X	X	
	Activities of daily living	- Modified Schwab and England scale	X	X	X	
	Demographics and lifestyle	- Medical history	X	–	–	
		- Lifestyle exposure	X	–	–	
		- Medication (Parkinson and other)	X	X	X	
		- Non-pharmacologic Treatments	X	X	X	
	Biospecimens	- Stool	X	X	X	
		- EDTA Plasma (DNA)	X	–	–	
		- PAX Gene (RNA)	X	X	X	
		- peripheral blood mononuclear cells (PBMCs)	X	–	–	
		- Plasma (no PBMC)	X	X	X	
		- Serum	X	X	X	
		- CSF	X	–	X	
	Autonomic function	- Holter ECG	X	X	X	
		- 12-lead ECG	X	X	X	
		- Blood pressure (standing and supine)	X	X	X	
	Brain structure and function	- Structural MRI (T1, T2, FLAIR, DTI, QSM)	X	–	X	
		- Functional MRI (resting state, task-related)	X	–	X	
Verily Study Watch	Physiological and environmental parameters	- PPG	Continuous data collection, up to 23 h a day.			
		- ECG				
		- IMU including 3-axis accelerometer and 3-axis gyroscope				
		- EDA				
		- Skin temperature				
		- Relative humidity				
		- Barometric pressure				
		- Ambient light level				
Self reported patient questionnaires	Neuropsychological symptoms	- BDI-II				X
		- QUIP-RS				X
		- Apathy scale				X
		- STAI				X
	Sleep disorders	- SCOPA- sleep				X
		- Epworth sleepiness scale				X
	Quality of life	- PDQ-39				X
		- SF-12				X
	Various	- MDS-UPDRS-II				X
		- SCOPA- aut				X
		- RBD questionnaire				X
		- Wearing-off questionnaire				X
		- New Freezing of Gait Questionnaire				X
		- Radboud Oral Motor Inventory				X
		- Screening Questionnaire on Visual Impairment				X
		- PASE				X
Self reported caregiver questionnaires	Burden	- CSI				X

*BDI* Beck Depression Inventory, *CSF* Cerebro-Spinal Fluid, *CSI* Caregiver Strain Index, *DNA* Deoxyribonucleic acid, *DTI* Diffusion Tensor Imaging, *ECG* electrocardiogram, *EDA* electrodermal activity, *EDTA* Ethylene Diamine Tetra Acid, *FLAIR* Fluid Attenuation Inversion recovery, *H&Y* Hoehn & Yahr, *IMU* Inertial Measurement Unit, *MDS-UPDRS-I/II/III/IV* Movement Disorders Society-sponsored revision of the Unified Parkinson’s Disease Rating Scale, non-motor experiences of daily living section (I), motor experiences of daily living section (II), motor examination section (III), and motor complications section (IV), *MMSE* Mini Mental State Examination, MoCa Montreal Cognitive Assessment, *MRI* Magnetic Resonance Imaging, *PASE* Physical Activity Scale for the Elderly, *PAX genes* Paired box genes, *PBMC* Peripheral Blood Mononuclear Cell, *PDQ-39* 39-item Parkinson Disease Questionnaire, *PPG* photoplethysmograph, *QSM* Quantitative Susceptibility Mapping, *QUIP-RS* Questionnaire for Impulsive-Compulsive Disorders in Parkinson’s Disease-Rating Scale, *RBD* Rapid eye movement sleep Behaviour Disorder, *RNA* Ribonucleic Acid, *SCOPA-aut* SCales for Outcomes in PArkinson’s disease, autonomic function section, *SCOPA-sleep* SCales for Outcomes in PArkinson’s disease, sleep section, *SF-12* 12-item Short Form, *STAI* State Trait Anxiety Inventory for Adults, *TUG* Timed Up and Go test

^a^Medical examinations & clinical assessments will be videotaped unless a subject declines; ^b^ ± 60 days; ^c^ within 4 weeks after each in-clinic visit

After each visit participants complete a set of validated questionnaires at home, including questionnaires about medication use, quality of life, lifestyle, neuropsychological symptoms, autonomic symptoms, sleep, and vision, among others [23–33]. These questionnaires are completed within 4 weeks after each visit, via an online survey module.

Finally, a copy of the medical record of the participant’s primary care physician is requested. This provides a rich information source of known and unknown markers of disease progression.

**Fig. 1:**
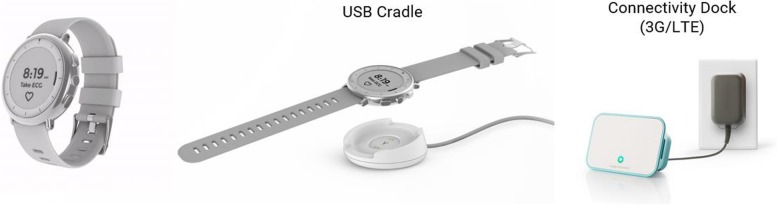
The Verily Study Watch (an Investigational Device), along with syncing/charging cradle and Study Hub. The photographs are owned by Verily and have a copyright. Verily kindly granted written permission to use and adopt if for this publication

Participants are asked to wear the Verily Study Watch preferably 24/7, except during charging, for 2 years. During the baseline visit, the trial assessor discusses the importance of ambulatory monitoring with the subject and explains and demonstrates the use of the Study Watch. Moreover, a paper-based user manual is made available to each participant. The Verily Study Watch is an investigational, non-CE marked, multi-sensor wearable device designed to extend data collection for clinical studies beyond trial sites and into the free-living environment. For its use in the Personalized Parkinson Project approval is obtained by the Dutch Health and Youth Care Inspectorate. The device enables the collection of physiological and environmental data about acceleration/orientation, pulse rate, electrodermal activity (EDA), electrocardiogram (ECG), barometric pressure, relative humidity, environmental temperature, and ambient light level. The Verily Study Watch is intended for passive data collection only, with minimal information communicated to the user via the device (Fig. 1). Data from the Verily Study Watch are encrypted and sent securely to the Verily cloud using a USB syncing/charging cradle and wireless connectivity bridge (the Verily Study Hub). The Study Watch has been deployed in several studies, mainly in the United States, including the Baseline Health Study [34] and the Parkinson Progression Markers Initiative (PPMI) [5].

### Subject retention strategies

Throughout the study, a dedicated helpdesk proactively assists participants, addresses problems and questions, and solves/communicates issues related to the execution of the trial. In addition to the study helpdesk, assigning a personal trial assessor to each participant during the entire study period is an important retention strategy. Participants are kept informed about the study with newsletters and participant events. In addition, the study website will be used for regular recruitment updates, combined with an extensive information package on topics that are found to be relevant for people with PD. The topics will be selected by the participants themselves.

### Quality management

All study procedures are being performed at a single centre, the Radboud university medical center. The study assessors were extensively trained and certified for the various elements of the protocol, before enrollment of the first study subject. Written standardized operating procedures (SOPs) were developed, detailing all data collection and study procedures. At least once a month, the study team discusses unexpected situations and problems encountered with execution of the study protocol. Frequent monitoring of the data quality ensures a timely identification of both systematic and incidental deviations. On a monthly basis an independent study monitor checks key elements of the study, e.g., inclusion rates, informed consents, data entry, protocol deviations, and (serious) adverse event reporting. If quality control issues emerge, the study protocol and SOPs will be amended and trial assessors will be retrained.

### Data management and protection to subject privacy

#### Data protection

For the PPP study, a Polymorphic Encryption and Pseudonymization (PEP) infrastructure was developed and is used to protect the data and the privacy of the participants [35]. For each participant, this method generates unique pseudonyms for the different participating research groups. The PEP was created to deal with the rigidity of the traditional encryption/decryption process by using polymorphic encryption right after collection and before storage. In that manner, there is no need to a priori fix in the encryption key for the data. The PEP system allows different researchers to have access to the entire dataset or only a subset of the dataset with a specific decryption key. In addition, the polymorphic encryption method avoids the combination of different datasets by different decryption key-holders, since each key only decrypts that subset of the data. Due to its additional security, the PEP is an ideal approach to manage sensitive personal data with reduced risk of a participant’s privacy being violated.

#### Data handling and storage

An electronic data capture and management system, Castor (https://data.castoredc.com), is used for the collection of most study data. This is a GCP-certified data management system with servers in accordance with the NEN7510 norm for information security in healthcare. All medical examinations and clinical assessment data are entered directly into Castor and stored in a pseudonymized manner. Similarly, Castor is used by subjects to complete the online questionnaires as part of the annual assessment. Holter ECG and 12-lead ECG data are also stored directly into Castor. MRI data is collected and stored at the Donders Center for Cognitive Neuroscience (DCCN), following their data management procedures: after data collection a backup is stored locally on the DCCN data servers; a copy is transferred to PEP, based on a PEP-generated pseudonym. Participating researchers can access data only via PEP. For the Verily Study Watch data, Verily will first preprocess the data and subsequently upload it to PEP. Verily only knows the device identifiers, but does not know the study participants.

All biospecimens are biobanked at the Radboud Biobank (Nijmegen, the Netherlands) [36], with their sample tracking system, sample processing system operation procedures, and standardized sample storage conditions being employed. In addition to Radboud’s laboratory capabilities, some sample processing will be performed by Verily Life Sciences. Verily has an on-site biobank as well as laboratories for molecular biology, chemistry, immunology, and systems biology including a population scale genomics facility. Verily’s laboratories use a laboratory information system for sample tracking, and all staff are trained on standard operation procedures for sample biobanking and processing. PEP pseudonyms for all samples have been added to both biobanks’ sample tracking systems.

### Statistical analyses

The primary objective of this study is to evaluate the rate of disease progression and, ultimately, to evaluate treatment response with respect to the presence of both established and potential biomarkers. More specifically, we will address two objectives in the short term:

1. The individual associations between potential biomarkers and disease progression in Parkinson’s disease, with progression being expressed in terms of rate of decline in two key outcome domains of the disease, i.e., motor functioning and cognitive functioning.
2. The prediction of disease progression after 1 year and 2 years on each of the two outcome domains.

Motor functioning will be determined using the score from the motor section (part III) of the Movement Disorders Society-sponsored revision of the Unified Parkinson’s Disease Rating Scale (MDS-UPDRS) [37], measured in the OFF state. Cognitive functioning will be determined using the score from the Montreal Cognitive Assessment scale (MoCA), a scale for global cognitive abilities that is validated for use in Parkinson’s disease [38].

For each of the two outcomes, the prediction will be made for absolute change scores (as a continuous measure) and clinically important change scores or clinically important threshold (as a dichotomous measure). For motor functioning, the dichotomous measure is a change of at least 5.5 [39, 40]. For cognitive functioning, the dichotomous measure is a follow-up score below 23.5 [41]. Both will be calculated over the first year of follow-up, as a measure for short-term disease progression (hence with direct clinical relevance for near-term treatment decisions), and over 2 years of follow-up, as a measure for mid-term prognosis.

The association between a potential biomarker and the change in the PD outcome (objective 1) will be evaluated with a multivariable linear model, adjusted for age and disease duration. For objective 2, a variety of different model building methods (e.g., forward stepwise logistic regression or lasso penalized regression methods) will be considered in the training set such that up to three candidate models may be assessed in the second half of the sample [42]. The training and testing set will be randomly selected from the entire sample. One of these models will be designated as primary for the formal assessment of objective 2 before the outcomes in the test set are unblinded. If the number of events using the split sample approach is insufficient to develop a strong model in the first half sample, cross-validation on the full sample will be considered as an alternative. For instance, as a rule of thumb, there should be no more than 1 predictor variable for every 10 events. Thus, before the data are analyzed, the list of variables to be considered may need to be trimmed.

If the selected predictors are available in the Parkinson Progression Markers Initiative (PPMI) dataset [5], this dataset will be used for external model validation. If this is not possible, at least internal validation will be performed using the test set and, if the model is refit on the full data, bootstrapping.

Additional examples of a priori hypotheses that we aim to address are, e.g.,:

- Activity levels extracted from the Verily Study Watch in the weeks preceding a clinic visit can predict Total and Part III scores on the MDS-UPDRS.
- Persons with higher levels of disease burden (including motor, affective, and cognitive symptoms) will show decreased heart rate variability when compared to persons with lower levels of disease burden.
- Neurocognitive testing scores predict the development of mild cognitive impairment [43, 44].
- Clinical motor progression in PD is the net outcome of the shifting balance between parietal hyperactivity (compensation) and basal ganglia hypoactivity (primary pathophysiology).

#### Sample size considerations

For the first objective, we will test the association between potential biomarkers and the deterioration of PD on each of the two outcome domains. In this test, both the dependent and independent variables are continuous measures. In order to adjust for multiple testing issues (up to 55 biomarkers and two outcome measures) we will use a two-sided significance level of 0.05/(2 × 55), i.e., 0.00045 instead of 0.05. In order to detect an association with a correlation coefficient of at least 0.2 with a power of 90%, 565 patients are needed. Taking into account an attrition rate of 10% and missing data for approximately 3% of patients, we aim to include 650 patients in total. If additional novel biomarkers are selected for model consideration prior to the initiation of analysis, power of 80% will be retained with 100 or fewer biomarkers considered.

For the second objective we will use the dichotomous outcomes to estimate the required sample size. We will use a split sample approach to develop the model in the first half of the dataset and test it on the second half of the data. Therefore, if there are a total of 565 patients evaluable, as dictated by the power calculation for the first objective, there will be 282 patients available to assess the accuracy of the model in the test set. It is desirable to demonstrate that the accuracy is greater than 70%, i.e., the probability that the model correctly predicts whether the patient did or did not have an event using a pre-specified threshold, is larger than 70%. We will have 80% power to demonstrate that the model is more accurate than 70% if the true accuracy is 77% or higher. If sensitivity or specificity at a single cut point is lower than desired for clinical utility, power may be improved by assessing the AUC (area under the ROC curve) rather than the accuracy at a single threshold.

## Discussion

The key purpose of the Personalized Parkinson Project is to contribute to the understanding of the differences in etiology, pathophysiology, phenotypic diversity, and disease progression among individual PD patients. Although previous cohort studies have largely contributed to elucidate the differences between PD patients and healthy control subjects [8, 45–47], we remain unable to understand the basis for the large diversity of phenotypes and variability in progression rates among PD patients. It remains unclear why some PD patients stay functional and independent long into the disease, while others progress to significant motor and/or cognitive impairment and are unable to live unassisted relatively early in the course of the disease. The 2-year follow-up captures inter-individual differences in the speed of disease progression on numerous clinically relevant outcomes, including motor- and non-motor symptoms and endpoints that are relevant in the earlier phases of the disease, such as the ability to work.

The PPP has several unique elements: an unbiased approach to patient selection, with purposely broad inclusion criteria (also allowing for presence of co-morbid conditions); repeated clinical, molecular, and imaging data collection performed at a single center; and multi-dimensional analysis to uncover novel biomarkers of PD. A broad biomarker definition will be applied, in line with the recently proposed modular set of biomarker assessments [48]. In addition, participants will be followed for 2 years using a wearable multi-sensor device, which creates the opportunity to continuously monitor aggregated features (e.g., daily and weekly activity level, mean daily pulse rate and its variability, average sleep efficiency per day) to characterize each participant over time. The very liberal inclusion criteria, where participants with co-morbidities are encouraged to join, allow us to collect data from “real-life” patients and explore how co-morbidities impair the overall expression of PD. Moreover, although we aim for a unique dataset, to the extent possible we have harmonized data collection with previously performed cohort studies including the Parkinson Progression Marker Initiative [5], Luxembourg Parkinson’s Study [11] and the Oxford Parkinson Discovery Cohort [12]. In this manner we also support initiatives such as the Critical Path for Parkinson’s Consortium (CPP), that aim to create integrated unified databases, thus allowing to increase sample sizes or add control populations and further enhance research.

Furthermore, the PPP cohort will contribute data managed through an unprecedented digital security system, which will allow sharing of data with researchers worldwide with maintenance of participants’ privacy. The digital security system is based on a multi-point, privacy-by-design strategy: (a) participants provide informed consent, also for the important element of data sharing; (b) signed contractual agreements with researchers are in place to ensure that no attempts towards de-identification or commercialization of the raw data will be attempted; (c) governance policies restrain access to the data to only qualified researchers; (d) an innovative pseudonymization and encryption process is applied [35]; and (e) a protected research environment is used to analyze the data.

Lastly, powered by Verily’s analytics capabilities, the PPP will allow us to address research questions of great scientific and clinical value to improve our understanding of PD pathology and variation between patients in terms of disease progression, therapy response (both efficacy and tolerance), and survival. Though we will not be able to adjust for normal age-related changes due to the lack of a matched control group, the PPP data will help to identify new biomarkers to predict differences in prognosis and treatment response between patients, an important step to improving existing treatments, developing new therapeutic approaches, and providing PD patients with a more precise and personalized disease management approach. Finally, this cohort will serve as a source of data for qualified researchers worldwide, allowing them to use their research capacity to further address and enhance the main aims of this study.

## Data Availability

The datasets generated during this study will be made available to qualified researchers worldwide, for which the participants provide informed consent. The Research and Data Sharing Review Committee (RDSRC) will protect subjects’ privacy by limiting the availability of the study data and controlling access to sources of information that might potentially be used to identify the individual subjects associated with the biospecimen analyses. Additional proposals to use biospecimens and/or accrued clinical and device data and data from the analysis of samples for research proposes will require additional review and approval from the RDSRC. Such approvals will also require subject re-consent and ethical approval, if the current ICF does not cover the proposed intended use. The RDSRC will assess the relevance and scientific quality of research proposals for which study data or material is requested. In addition, a Data Safety and Monitoring Committee (DSMC) has been established, as required under Dutch law. The DSMC safeguards the interests of trial participants by monitoring outcomes and efficacy as they pertain to safety.

## Abbreviations

AUC: Area Under the Curve
BDI: Beck Depression Inventory
CSF: Cerebro-Spinal Fluid
CSI: Caregiver Strain Index
DCCN: Donders Center for Cognitive Neuroscience
DNA: DeoxyriboNucleic Acid
DTI: Diffusion Tensor Imaging
ECG: Electro CardioGram
EDA: Electro Dermal Activity
EDTA: Ethylene Diamine Tetra Acid
FLAIR: FLuid Attenuation Inversion Recovery
GCP: Good Clinical Practice
H&Y: Hoehn & Yahr
IMU: Inertial Measurement Unit
MDS-UPDRS-I/II/III/IV: Movement Disorders Society-sponsored revision of the Unified Parkinson’s Disease Rating Scale, non-motor experiences of daily living section (I), motor experiences of daily living section (II), motor examination section (III), and motor complications section (IV)
MMSE: Mini Mental State Examination
MoCa: Montreal Cognitive Assessment
MRI: Magnetic Resonance Imaging
PASE: Physical Activity Scale for the Elderly
PAX genes: Paired box genes
PBMC: Peripheral Blood Mononuclear Cell
PD: Parkinson’s disease
PDQ-39: 39-item Parkinson Disease Questionnaire
PEP: Polymorphic Encryption and Pseudonymization
PPG: photoplethysmograph
PPMI: Parkinson’s Progression Marker Initiative
PPP: Personalized Parkinson Project
QSM: Quantitative Susceptibility Mapping
QUIP-RS: Questionnaire for Impulsive-Compulsive Disorders in Parkinson’s Disease-Rating Scale
RBD: Rapid eye movement sleep Behaviour Disorder
RNA: Ribonucleic Acid
ROC: Receiver Operating Characteristic
SCOPA-aut: SCales for Outcomes in PArkinson’s disease, autonomic function section
SCOPA-sleep: SCales for Outcomes in PArkinson’s disease, sleep section
SF-12: 12-item Short Form
SOP: Standardized Operating Procedure
STAI: State Trait Anxiety Inventory for Adults
TUG: Timed Up and Go test
